# HIV infection drives IgM and IgG3 subclass bias in *Plasmodium falciparum*-specific and total immunoglobulin concentration in Western Kenya

**DOI:** 10.1186/s12936-019-2915-7

**Published:** 2019-08-30

**Authors:** Eliud O. Odhiambo, Dibyadyuti Datta, Bernard Guyah, George Ayodo, Bartholomew N. Ondigo, Benard O. Abong’o, Chandy C. John, Anne E. P. Frosch

**Affiliations:** 1 Department of Biomedical Science and Technology, Maseno University, Maseno, Kenya; 2 Center for Global Health Research, Kenya Medical Research Institute, Kisumu, Kenya; 3 Department of Pediatrics, Indiana University School of Medicine, Indianapolis, USA; 4 Jaramogi Oginga Odinga University of Science and Technology, Bondo, Kenya; 5 Department of Biochemistry and Molecular Biology, Egerton University, Nakuru, Kenya; 6 Laboratory of Malaria Immunology and Vaccinology, National Institute of Allergy and Infectious Disease, NIH, Bethesda, MD USA; 7 Department of Biology, Faculty of Science and Technology, National University of Lesotho, Roma, Lesotho; 8 Department of Medicine, University of Minnesota, Minneapolis, USA; 9 Hennepin Healthcare Research Institute, Minneapolis, MN USA

**Keywords:** HIV, *Plasmodium falciparum*, Antibodies, Immune activation, Viral load, CRP, B cells, Malaria

## Abstract

**Background:**

HIV infection is associated with more frequent and severe episodes of malaria and may be the result of altered malaria-specific B cell responses. However, it is poorly understood how HIV and the associated lymphopenia and immune activation affect malaria-specific antibody responses.

**Methods:**

HIV infected and uninfected adults were recruited from Bondo subcounty hospital in Western Kenya at the time of HIV testing (antiretroviral and co-trimoxazole prophylaxis naïve). Total and *Plasmodium falciparum* apical membrane antigen-1 (AMA1) and glutamate rich protein-R0 (GLURP-R0) specific IgM, IgG and IgG subclass concentrations was measured in 129 and 52 of recruited HIV-infected and uninfected individuals, respectively. In addition, HIV-1 viral load (VL), CD4^+^ T cell count, and C-reactive protein (CRP) concentration was quantified in study participants. Antibody levels were compared based on HIV status and the associations of antibody concentration with HIV-1 VL, CD4^+^ count, and CRP levels was measured using Spearman correlation testing.

**Results:**

Among study participants, concentrations of IgM, IgG1 and IgG3 antibodies to AMA1 and GLURP-R0 were higher in HIV infected individuals compared to uninfected individuals (all *p *< 0.001). The IgG3 to IgG1 ratio to both AMA1 and GLURP-R0 was also significantly higher in HIV-infected individuals (*p* = 0.02). In HIV-infected participants, HIV-1 VL and CRP were weakly correlated with AMA1 and GLURP-R0 specific IgM and IgG1 concentrations and total (not antigen specific) IgM, IgG, IgG1, and IgG3 concentrations (all *p *< 0.05), suggesting that these changes are related in part to viral load and inflammation.

**Conclusions:**

Overall, HIV infection leads to a total and malaria antigen-specific immunoglobulin production bias towards higher levels of IgM, IgG1, and IgG3, and HIV-1 viraemia and systemic inflammation are weakly correlated with these changes. Further assessments of antibody affinity and function and correlation with risk of clinical malaria, will help to better define the effects of HIV infection on clinical and biological immunity to malaria.

## Background

Despite the fact that human immunodeficiency virus (HIV) infection is largely considered a disease of altered intracellular immunity, it has also been shown to have a major impact on the B cell compartment. Early on, this primarily included observations of high rates of B cell lymphomas, hypergammaglobulinemia, and autoantibodies in the setting of HIV infection [1]. Over the years, there has been a growing body of evidence that there are also alterations in B cell function. HIV infection leads to impaired immune responses to vaccines and infections including pneumococcal disease [2–6]. Considerable research has focused on the mechanism behind these impairments in B cell immunity. Diminished T follicular helper cell support, altered responsiveness to T cell stimulation, and intrinsic B cell dysfunction have all been considered as a cause of these impairments and recently phenotypically exhausted B cells have been described in the setting of HIV infection [1, 7–11]. However, these B cell phenotypic changes do not fully explain why there is clinically impaired B cell immunity, particularly in cases where antigen specific antibody responses are maintained despite coexisting B cell phenotypic abnormalities, as has been described with malaria and HIV coinfection [12].

For individuals living in sub-Saharan Africa, the epicenter of the HIV epidemic, malaria is a major cause of morbidity and mortality [13]. Although co-trimoxazole prophylaxis has been shown to provide some protection against malaria [14], interaction between HIV and malaria could have a significant impact the health of infected individuals in regions with low access to HIV care [15, 16]. This is particularly true as malaria endemic countries face shortages of co-trimoxazole and public health officials consider scaling back its use among people living with HIV. Indeed, HIV has been linked with increased malaria risk and severity [17, 18].

B cell responses are a key component of adaptive immunity to malaria and past studies have associated antibodies against pre-erythrocytic and blood stage malaria antigens with clinical malaria protection [19–21]. The mechanism behind HIV mediated vulnerability to malaria is not clear. Overall IgG concentrations to several malaria antigens including AMA1 and merozoite surface protein (MSP) appear to be maintained if not elevated in HIV infection, although there is evidence using serological protein microarray assays that the breadth of malaria antibody responses may be decreased [12, 22–24]. Similarly, in the population described in this study, malaria specific IgG levels to a panel of *Plasmodium falciparum* antigens tended to be higher in HIV infected individuals compared to HIV uninfected individuals [12]. Antibody effector function is highly dependent on the Fc portion of the antibody [25], and understanding the distribution of malaria specific antibodies among classes and subclasses may lend some insight on the impact of HIV infection on antibody function.

To determine whether HIV alters the concentrations of malaria specific antibody classes and subclasses, the levels of IgM, IgG and IgG1–4 specific to two *P. falciparum* protein antigens, AMA1 and GLURP-R0, were measured in plasma of HIV-infected and uninfected participants living in western Kenya. Because both antiretrovirals and cotrimoxazole can affect malaria vulnerability, this was done at the point of HIV testing. Based on previous finding that a difference in IgG3:IgG1 ratio is associated with persistent clinical malaria risk in both stable and unstable malaria transmission areas [26], changes in the IgG3:IgG1 ratio between HIV-infected participants and uninfected participants was compared. Total IgM, IgG and IgG1–4 levels was measured to determine if the trends of malaria-specific antibody concentrations were mirrored in total (non-malaria specific) immunoglobulin (Ig) concentrations, i.e. was this an antigen specific or global phenomenon. Finally, in order to understand the influence of markers of HIV immunodeficiency and immune activation (HIV-1 VL, CD4^+^ counts and CRP) on malaria-specific antibodies, the concentrations of these markers were correlated with these antibody levels.

## Methods

### Study participants, area and design

This was a cross sectional study designed to evaluate the effects of HIV infection on malaria immunity, as previously reported, at Bondo Sub-County Hospital, Siaya county, western Kenya [12]. Bondo District lies between an altitude of 0° 26° to 0° 90° and from longitude 33° 58° E and 34° 35° W and is among the malaria holoendemic regions in Kenya. Patients of 18 years of age or older undergoing HIV testing at Bondo Sub County Hospital were eligible for recruitment into the study. Exclusion criteria included pregnancy, current antimalarial use, acute illness (including fever), and chronic illness (other than HIV) or medication use that may affect immune responses. This study recruited 190 eligible patients (138 HIV-infected and 52 uninfected participants) who made an informed consent to participate. Approximately 40 mL venous blood was collected from volunteers into sodium heparinized vacutainer. Venous blood was separated using density gradient centrifugation (Ficoll Histopaque, Sigma-Aldrich, St. Louis, Missouri) to obtain plasma and peripheral blood mononuclear cells (PBMCs). Specimens were processed within 6 h of collection and plasma and PBMCs were stored in − 20 °C freezer and liquid nitrogen, respectively. In addition, dried blood spots (DBS) were collected for viral load testing. More individuals with HIV infection were enrolled so that the study would have the power to detect differences in serologic outcomes within subgroups of the HIV infected participants, specifically individuals with CD4 counts above and below 200. Approximately one HIV-uninfected participant was recruited after every three HIV-infected participants throughout collection period. This alternating recruitment pattern was employed so that HIV infected and uninfected individuals would be spaced evenly throughout the collection period of 5 months (from May to October, 2012). Out of the 190 samples collected, antibody levels were tested in the first 181 participants enrolled (129 and 52 HIV-infected and uninfected, respectively) based on specimen and resource availability.

### CD4^+^ T cell count, HIV-1 viral load, and C-Reactive Protein (CRP) concentration testing

Absolute CD4^+^ counts were obtained by FACSCount system (BD Biosciences, San Jose, CA [27]. This was done at the Bondo clinical laboratory which undergoes regular internal and external quality control audits as prescribed by the Kenyan Ministry of Health. Briefly, 50 µL of whole blood was pipetted into tube containing CD4/CD3 reagents conjugated with PE and Cy5 dye (PE-Cy5) fluorescence respectively and vortexed upright for 6 s. This was followed by 80 min incubation at room temperature before adding 50 µL of fixative solution. This was run on a FACSCount system and CD4^+^ counts for samples were obtained using FACSCount CD4 software.

HIV-1 viral load was measured by the Centers for Disease Control and Prevention (CDC)-Kenya core lab from dried blood spots as described using the Abbott m2000rt real-time system [28, 29]. Two discs were punched from DBS and incubated in 50 mL conical tube containing a proprietary Abbott mLysis_DNA_ buffer. This was followed by incubation for 2 h at room temperature while mixing before conveying it to an Abbott reaction vessel. RNA was isolated using the Abbott *m*Sample Preparation System_*DNA*_ which utilizes magnetic particles for nucleic acid binding, washing and extraction. The extracted RNA was amplified on Abbott Optical Reaction Plate after mixing the RNA with kit reagents (HIV-1 oligonucleotides, polymerase enzyme, and proprietary activation reagent) for amplification with real-time PCR. This kit converts viral RNA to cDNA via a thermostable recombinant *Thermus thermophilus* DNA polymerase. The target sequence for this assay is in the highly conserved *pol integrase* region of the HIV-1 genome. Each reaction contains an internal control, an unrelated RNA product. The amplification products were fluorescently detected and converted to viral load by the m2000rt real-time analyser (Abbott Laboratories, Abbott Park, IL) using a set of calibrators of known RNA concentration.

CRP concentrations were determined by standard ELISA per kit instructions as previously reported using a human CRP ELISA Kit (Millipore Corporation, Darmstadt, Germany) [12, 30, 31]. The stock standard (1000 ng/mL) was subjected to 1:100 dilution using wash buffer (provided by the manufacturer) to obtain the first standard (10 ng/mL) which was subjected to four threefold serial dilutions to produce a total of five standards with the fifth standard having a concentration of 0.12 ng/mL. Samples and controls were diluted systematically in wash solution to obtain a dilution of 1:4000. Diluted standards, controls, and samples (100 µL) were dispensed into plate and incubated at room temperature (20–25 °C) for 30 min. This was followed by 5 times wash with wash solution then 100 µL conjugate solution (anti-human CRP antibody) was added and incubated at room temperature for 30 min. The plate was washed again 5 times using wash solution after which 100 µL substrate solution was added and incubated for 10 min at room temperature. Stop solution (100 µL) was added and plate reaction read at 450 nm (Molecular Devices, Sunnyvale, CA). CRP plasma concentrations were calculated as per manufactures instructions. The manufacturer reported intra- and inter assay coefficients of variation (CVs) of up to 4.6 and 6.0%, respectively. Duplicate testing was done with 10% of samples with a median inter assay CVs for duplicate measurements of CRP was 20.5%. For duplicate samples an average value was used for analysis.

### Malaria-specific and total immunoglobulin testing

IgM, IgG and IgG1–4 antibodies against *P. falciparum* antigens apical membrane antigen-1 (AMA1, full length ectodomain, 3D7) and glutamate rich protein (GLURP, conserved non-repeat N-terminal region, amino acids 25-514, R0) antigens were tested. AMA1 and GLURP-R0 are vaccine candidates and were chosen based on antigen availability and their association with protection from clinical malaria in a number of population-based studies in malaria endemic areas [32–37].

A modified ELISA protocol was used as previously described [38–41] for testing malaria-specific IgG and IgM antibody levels. Briefly, recombinant AMA-1 and GLURP0-R0 antigens were diluted in 1xPBS (0.1 µg/µL) and 50 µL per well was used to coat 96 well Immunol 4 (Thermo Labsystems # 3855, US) plates at 4 °C overnight. The plates were washed three times with 1× PBS/Tween 20 and then blocked with 5% Blotto (and Seablock diluent buffer for IgM ELISA) for 1 h at room temperature. The plates were again washed three times with 1× PBS/Tween 20 followed by addition of 50 µL of tested samples, negative controls (North American controls, NACs) and positive controls (pool of plasma samples from Ugandan malaria positive patients) diluted (1:100) in 5% Blotto (and Seablock diluent buffer for IgM ELISA) into each well. The plates were then incubated for 2 h at room temperature. The plates were washed three times wash with 1× PBS/Tween 20. This was followed by adding 50 µL goat anti-human IgG-Alkaline Phosphatase diluted (1:1000) in Blotto (and goat anti-human IgM-Alkaline Phosphatase subjected to 1:4000 dilution in Seablock diluent buffer for IgM) after another three times washes with 1× PBS/Tween 20 and incubated for 1 h at room temperature. Wells were washed six times washes with 1× PBS/Tween 20 and substrate was added for a 30 min incubation in the dark. Reaction was stopped by addition of 3 N NaOH and OD value read at 405 nm (Molecular Devices, Sunnyvale, CA).

For IgG subclass testing, recombinant AMA-1 and GLURP0-R0 antigens were diluted in 1xPBS (0.1 µg/µL) and 50 µL of this solution was used to coat 96 well Immunol 4 (Thermo Labsystems # 3855, US) plates at 4 °C overnight. The plates were washed three times with 1× PBS/Tween 20 then blocked with blocking buffer (PBS/3% BSA) for 1 h at room temperature. Again, the plates were washed three times with 1× PBS/Tween 20. 50 µL of tested samples, negative controls (North American controls, NACs) and positive controls (pool of plasma samples from Ugandan malaria positive patients) diluted (1:100) in diluent buffer (PBS/1% BSA) were added to each well. After an overnight incubation at 4 °C, the plates were washed three times with 1× PBS/Tween 20. 50 µL of secondary biotinylated antibodies (mouse anti-human IgG 1–4 biotinylated antibodies) diluted 1:1000 in diluent buffer (PBS/1% BSA) was added and incubated for 45 min at room temperature. The plates were washed three times wash with 1× PBS/Tween 20. Streptavidin conjugated alkaline phosphatase diluted 1:2000 in diluent buffer (PBS/1% BSA) was added (50 µL/well) and plates were incubated at room temperature for 30 min. The plates were washed six times wash with 1× PBS/Tween 20. Substrate (50 µL/well) was added and plates were incubated in dark for 20 min. Reaction was stopped by addition of 3 N NaOH and OD value read at 405 nm (Molecular Devices, Sunnyvale, CA). 19% of samples were done in duplicate and had a median optical density inter- and intra-assay CVs of 9.1 (range 5.3–18.7) and 5.1 (range 1.9–18.8), respectively (Additional file 1: Table S1).

Optical density (OD) from ELISA tests were exported into excel file for the calculation of arbitrary units (AU) using ODs obtained from negative controls (North American Controls, NACs). NACs were known never to have been exposed to malaria based on travel history. Quantitative antibody levels were expressed in AU by dividing test sample’s optical density (OD) by the sum of mean OD and 3 standard deviations (SD) of negative controls (NACs) as previously described [26]. AU values ≥ 1 were considered responders or seropositive to the respective antigen.

Total immunoglobulin concentrations were tested by Bio-Plex Pro using premixed multiplex kit for detecting total human immunoglobulins IgG1, IgG2, IgG3, IgG4 and lgM (Bio-Rad). Total IgG concentration was obtained by eBioscience human IgG total ELISA kit (ThermoFisher). Preparation and reading of the Bio-Plex and ELISA assay kits were done in accordance to manufacturer’s instructions. For Bio-Plex assay, 50 µL of diluted beads were added into each well followed by two washes using 100 µL Bio-Plex wash buffer (provided). The provided standard was reconstituted by adding 781 µL diluent solution and subjected to fourfold dilutions to obtain a total of 8 standards. Samples and controls were diluted in diluent solution (provided) to obtain a dilution of 1:40,000. 50 µL of vortexed samples, standards, blank and controls added into plate. The plate was covered (using aluminium foil) and incubated for 1 h at room temperature (RT) while shaking at 850 revolutions per minute (rpm). The plates were washed three times with 100 µL Bio-Plex wash buffer then 25 µL of detection antibodies added and incubated for 30 min at room temperature while shaking at 850 rpm in dark. This was followed by three times wash with 100 µL Bio-Plex wash buffer then 50 µL of 1× streptavidin-PE (SA-PE) added and incubated at room temperature for 10 min. Again, the plates were washed three times with 100 µL Bio-Plex wash buffer followed by suspension of beads in 125 µL assay buffer. Reading of the plates was done in accordance to manufacturers setting instructions. A standard curve was constructed and read off values for tested samples obtained as antibody concentration in ng/mL.

### Data analysis

Comparisons of antibody levels to AMA1 and GLURP-R0 (in arbitrary units) by HIV status, CD4^+^ level (< 200 vs ≥ 200) and VL (< 10,000 vs ≥ 10,000) and total immunoglobulin levels by HIV status were done using the Mann–Whitney test, and proportions of individuals with antibodies to AMA1 and GLURP-R0 according to HIV status were compared using the Chi squared test. Non-antigen specific immunoglobulin levels are referred to as *total* IgG, IgM or IgG subclass antibodies in this study. Correlations of antibodies with CRP, CD4^+^ counts and HIV-1 VL were obtained by the Spearman correlation test. Correlation coefficients between 0.1 and 0.3 were considered weak correlations as previously described [42]. Statistical analyses were done using STATA version 14.2 (Stata Corporation, College Station, TX, USA) and GraphPad Prism 7.0 (GraphPad Software Inc., CA, USA). For all analyses, *p*-values ≤ 0.05 were considered significant.

## Results

### Participants’ clinical characteristics

Malaria-specific antibody concentrations were measured in the first 181 individuals enrolled in the study (52 HIV negative and 129 HIV positive) living in Bondo Sub-County and obtaining medical care from Bondo Sub-County Hospital. Bed net use data was missing for 3 participants. CD4 data was missing from 1 participant secondary to lab equipment outage. Viral load data was missing from 1 participant. Average age was 3.8 years higher in HIV-infected individuals (*p *< 0.01, Table 1) [12]. There was a similar distribution between the two groups in gender and malaria infection status.

**Table 1:** Clinical characteristics of study participants according to HIV infection status

	HIV-negative	HIV-positive	p value
Number of participants (N)	52	138	
Age, median (IQR)	24.6 (21.6, 32.2)	29.4 (25.3, 36.2)	*< 0.001*
Female sex, N (%)	25 (48.1)	84 (60.9)	0.11
Malaria Positive, N (%)	3 (5.8)	12 (8.6)	0.55
Bed net use, N (%)	43 (84.3)^a^	107 (84.3)^b^	0.99
CRP, g/dL, median (IQR)	0.52 (0.27, 1.15)	4.72 (0.867, 26.12)	*< 0.0001*
CD4, cells/mL, median (IQR)	–	301 (180, 476)^c^	–
CD4 < 200, N (%)	–	42 (30.7)	–
HIV-1 Viral load, copies/mL, median (IQR)	–	50,370 (14,546–198,155)^c^	–

*p*-value comparing HIV-negative vs. HIV-positive participants from Chi square for proportions (female sex, malaria positive, CD4^+^ counts < 200), and Mann–Whitney test for medians (CRP concentrations and age)

Statistically significant differences (p < 0.05) are displayed in italics

*IQR* interquartile range: 25th percentile–75th percentile

^a^N = 51

^b^N = 136

^c^N = 137

### Comparison of antibodies in HIV-infected and uninfected individuals

**Fig. 1:**
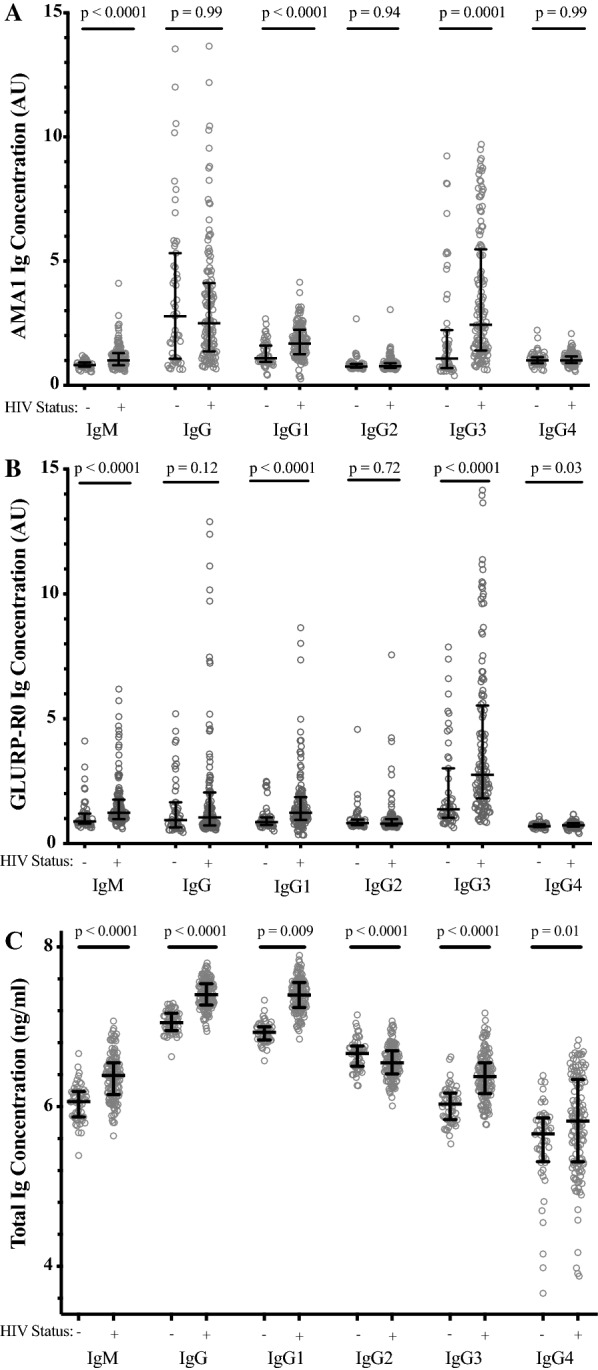
IgG, IgM and IgG subclass antibody concentration to **a** *P. falciparum* antigen AMA-1, **b** *P. falciparum* antigen GLURP-R0 and **c** total immunoglobulin concentration in study participants, according to HIV infection status. p values obtained from Wilcoxon rank-sum testing. HIV− N = 52, HIV+ N = 129

**Fig. 2:**
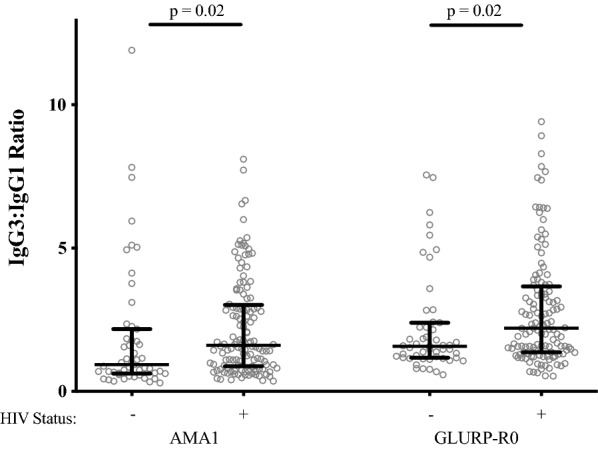
AMA1 and GLURP-R0 IgG3:IgG1 ratios in HIV-infected vs. uninfected participants. *P* values obtained from Wilcoxon rank-sum testing. HIV− N = 52, HIV+ N = 129

IgM, IgG1 and IgG3 antibody levels to both AMA1 and GLURP-R0 were significantly higher in HIV-infected participants compared to HIV-uninfected participants (*p *< 0.01, Fig. 1a and b). Total IgG, IgG2 and IgG4 antibody levels to AMA-1 and GLURP-R0 were not elevated in HIV-infected individuals except for IgG4 against GLURP-R0 (*p* = 0.03, Fig. 1a and b). Correspondingly, the proportions of serologic reactivity to AMA1 and GLURP-R0, defined as persons with an antibody concentration greater than 1 arbitrary unit, were also higher for IgM, IgG1 and IgG3 in HIV-infected participants compared to HIV-uninfected participants (*p *< 0.01, Table 2). IgG3:IgG1 ratios against both AMA1 and GLURP-R0 were higher in HIV-infected as compared to HIV-uninfected participants (*p *= 0.02, Fig. 2). It should be noted that these ratios are generated using arbitrary units for each subclass, so that a ratio of > 1 does not mean that the concentration per mL of blood of IgG3 is higher than IgG1. For the total immunoglobulin pool (not antigen specific), IgG, IgM, IgG1, IgG3 and IgG4 levels were higher in HIV-infected participants (*p *< 0.01, Fig. 1c), while total IgG2 levels were deficient in HIV-infected participants (*p *= 0.009, Fig. 1c). Both malaria-specific and total antibodies showed similar patterns when HIV-infected compared with uninfected participants (Fig. 1a–c).

**Table 2:** Proportion of study participants with a reactive IgG, IgM or IgG subclass antibodies to *P. falciparum* antigens AMA-1 and GLURP-R0, according to HIV infection status

Recombinant malaria antigens	HIV negative (N = 52)	HIV positive (N = 129)	p value^a^
IgM-AMA1, n (%)	7 (13)	65 (50)	*< 0.001*
IgM-GLURP-R0, n (%)	21 (40)	94 (73)	*< 0.001*
IgG-AMA1, n (%)	41 (79)	115 (89)	0.07
IgG-GLURP-R0, n (%)	24 (46)	71 (55)	0.28
IgG1-AMA1, n (%)	31 (60)	117 (91)	*< 0.001*
IgG1-GLURP-R0, n (%)	14 (27)	90 (70)	*< 0.001*
IgG2-AMA1, n (%)	6 (12)	24 (19)	0.25
IgG2-GLURP-R0, n (%)	12 (23)	30 (23)	0.98
IgG3-AMA1, n (%)	28 (54)	114 (88)	*< 0.001*
IgG3-GLURP-R0, n (%)	42 (81)	124 (96)	*0.001*
IgG4-AMA1, n (%)	27 (52)	63 (49)	0.71
IgG4-GLURP-R0, n (%)	1 (2)	2 (2)	0.86

Statistically significant differences (p < 0.05) are displayed in italics

^a^χ^2^ test

### Correlation of *P. falciparum*-specific antibodies with markers of HIV disease severity and immune activation

In HIV-infected individuals, total IgM, IgG, IgG1 and IgG3 showed weak positive correlation with both HIV-1 viral load and CRP concentration (*rho* values 0.21–0.36, *p* < 0.05, Table 5). There was no correlation between CD4 count and the total immunoglobulin levels (Table 5). Similar trends were seen among malaria specific antibody concentrations to AMA1 and GLURP-R0 with weak correlations of HIV-1 viral load and CRP with IgM and IgG1 concentration (Tables 3 and 4). Correlations were visualized with scatter plots of malaria-specific antibodies against VL and CRP (Additional file 1: Figure S1). Malaria specific IgM and IgG1 levels were not correlated with CD4 counts. Finally, there was no significant correlation between IgG3:IgG1 ratios with HIV-1 viral load or CD4^+^ and CRP levels (Tables 3, 4, and 5). Similarly, IgG3:IgG1 ratios against both AMA1 and GLURP-R0 were not different among those who met CD4 count criteria for acquired immunodeficiency syndrome (AIDS, CD4^+^ count < 200) and those with CD4^+^ counts ≥ 200 (Additional file 1: Figure S2).

**Table 3:** Correlation of antibodies to AMA1 with viral load (VL), CD4 count and C-reactive protein (CRP) concentration in HIV-infected individuals

Marker	N	IgM		Total IgG		IgG1		IgG3		IgG3:IgG1	
		Rho^a^	p value^a^	Rho^a^	p value^a^	Rho^a^	p value^a^	Rho^a^	p value^a^	Rho^a^	p value^a^
VL	128	*0.31*	*< 0.01*	− *0.19*	*0.04*	*0.21*	*0.02*	0.05	0.57	− 0.10	0.28
CD4	128	− 0.12	0.17	*0.26*	*< 0.01*	> − 0.01	0.97	0.04	0.64	0.11	0.21
CRP	129	*0.22*	*0.01*	− 0.03	0.71	*0.20*	*0.02*	0.17	0.052	0.08	0.39

Statistically significant differences (p < 0.05) are displayed in italics

^a^Spearman correlation test

**Table 4:** Correlation of antibodies to GLURP-R0 with viral load (VL), CD4 count and C-reactive protein (CRP) concentration in HIV-infected individuals

Marker	N	IgM		Total IgG		IgG1		IgG3		IgG3:IgG1	
		Rho^a^	p value^a^	Rho^a^	p value^a^	Rho^a^	p value^a^	Rho^a^	p value^a^	Rho^a^	p value^a^
VL	128	*0.21*	*0.02*	0.10	0.28	*0.24*	*0.01*	0.14	0.12	− 0.07	0.40
CD4	128	0.02	0.81	− 0.06	0.48	− 0.08	0.39	0.06	0.52	0.11	0.19
CRP	129	*0.18*	*0.047*	0.15	0.10	*0.25*	*< 0.004*	0.16	0.07	0.03	0.77

Statistically significant differences (p < 0.05) are displayed in italics

^a^Spearman correlation test

**Table 5:** Correlation of total immunoglobulins with viral load (VL), CD4 count and C-reactive protein (CRP) concentration in HIV-infected individuals

Marker	N	IgM		Total IgG		IgG1		IgG3		IgG3:IgG1	
		Rho^a^	p value^a^	Rho^a^	p value^a^	Rho^a^	p value^a^	Rho^a^	p value^a^	Rho^a^	p value^a^
VL	128	*0.36*	*< 0.0001*	*0.29*	*0.001*	*0.27*	*0.002*	*0.21*	*0.02*	0.05	0.61
CD4	128	− 0.12	0.17	0.03	0.70	0.01	0.90	− 0.10	0.26	− 0.14	0.11
CRP	129	*0.32*	*0.0002*	*0.28*	*0.002*	*0.32*	*0.0002*	*0.28*	*0.001*	0.08	0.38

Statistically significant differences (p < 0.05) are displayed in italics

^a^Spearman correlation test

## Discussion

The data presented here demonstrates that HIV-infected individuals living a highly malaria endemic region of Kenya have higher levels of IgM, IgG1 and IgG3 antibodies to the *P. falciparum* antigens AMA1 and GLURP-R0 when compared to HIV-uninfected individuals. This same pattern is seen in the total immunoglobulin pool. Although both IgG1 and IgG3 levels rise, there is a proportionally larger increase in IgG3 (increased IgG3:IgG1 ratio) to the malaria antigens in the setting of HIV infection. Among HIV infected individuals, increases in IgG1, IgM and to AMA1 and GLURP-R0 weakly correlate with HIV-1 viral load and CRP level, suggesting that HIV viraemia and inflammation may contribute to the observed changes. Together, these findings suggest that HIV related global inflammation could influence class switching for non-HIV antigens, in this case *P. falciparum* proteins.

As mentioned previously, clinical observations suggest that individuals with HIV are more susceptible to malaria infection and disease [18, 43, 44]. Further, existing research on immunoglobulin subclass distribution and malaria vulnerability in HIV-uninfected individuals suggest that subclass should be considered as a mediator of this clinical observation. Specifically, IgG1 and IgG3 concentrations to various malaria antigens have been correlated with malaria disease protection [26, 45–47]. While direct correlation between protection and antibody levels are beyond the scope of this study, it is notable that for IgM, IgG1 and IgG3, the median level to malaria proteins AMA1 and GLURP-R0 are *higher* in HIV infected participants, a group typically understood to be at increased risk for malaria. The presented data is evidence that deficiency of a particular subclass may not be a major driver of malaria disease vulnerability in HIV. It is possible that an increased class or subclass level could enhance malaria vulnerability in HIV. There is not direct evidence of this in this study, but increased IgM concentration has been implicated in *P. falciparum* immune evasion, although in a non-antigen specific manner, via Fc binding by infected red blood cells and rosette formation [48].

It is possible that the conclusions presented here are incorrect and malaria specific antibody subclass concentration *is* important in malaria protection in HIV, but this study was unable to detect it based on the study design [49–51]. In the literature, the biggest difference in HIV mediated malaria susceptibility has been described in areas of low malaria transmission [42]. This study was conducted in a highly endemic area for malaria based on the hypothesis that the population would have a more uniform malaria exposure history [52]. However, it is possible that a study in a low transmission area or in may reveal significant subclass deficiencies among HIV infected individuals.

HIV infected individuals in this study did not have a deficiency or imbalance toward malaria specific non-cytophilic (IgG2 and IgG4) antibodies, as has been seen in some populations with low malaria transmission and decreased clinical immunity [26]. Total IgG2 deficiency was found in this cohort similar as has been previously described [53] and interestingly IgG2, which primarily targets polysaccharide antigen, is considered important in pneumococcal immunity, a major cause of morbidity and mortality among people living with HIV in Africa [54, 55].

Although the relationship between B cell exhaustion (increased atypical B cells) and sublass production is unknown, previously reported findings that malaria specific atypical memory B cells are increased in HIV-infected individuals [12] suggest that these cells should be further studied as a source of subclass skewing. In the setting of malaria infection alone, Obeng-Adjei et al. have described increased Tbet expression in atypical B and correlated this with increased skewing to IgG3 subclass expression [56]. There is considerable evidence that these atypical B cells are present in several diseases with a high degree of inflammation—malaria, HIV, autoimmune diseases. However, it is not clear if observed B cell changes in these diseases are driven by chronic antigen stimulation through T cell receptor (TCR) and B cell receptor (BCR) signaling pathways, or if non TCR and BCR driven signals are important in B cell dysfunction in the setting of chronic inflammation. The observation that HIV-mediated inflammation is associated with subclass distribution among B cells of varying antigen specificity is suggestive that a mechanism outside of direct TCR and BCR signaling may be playing a role. This hypothesis is supported by multiple studies that describe a high degree of phenotypic exhaustion in the B cell compartment, which indicates that these abnormalities are not purely confined to B cells responding to the chronic antigen stimulus [9, 12, 49].

Even in mouse studies, the precise mechanisms that determine a B cell’s subclass are not clear. One possible mechanism to consider based on the presented findings stems from a unique aspect of HIV pathophysiology, which is directly relevant to B cell responses. T follicular helper cells, the primary T cells that drive the germinal center responses, are preferentially spared from cytotoxic T cell killing of HIV infected cells, leading to expansions of Tfh in lymphoid tissue [57]. However, although there are expansions of this population, Tfh are also preferentially infected with the HIV virus and have associated phenotypic changes [58, 59], and may lead to altered GC physiology and consequently class switching. Mechanisms outside of the GC may also be involved. There is considerable evidence that antigen-presenting cells have altered function in the setting of HIV including increased productions of B cell Activating Factor [60]. There is also evidence that direct interactions between plasmacytoid dendritic cells and HIV gp120 can impair TLR9 based B cell IFN-alfa signaling [61].

## Conclusions

In conclusion, this study demonstrates that total and malaria-specific IgM, IgG1 and IgG3 concentration and IgG3:IgG1 ratios are elevated in HIV-infected as compared to HIV-uninfected individuals. Further, some of these changes, specifically IgM and IgG1 levels, demonstrate a weak positive correlation with HIV viral load and CRP level. This study only investigated the levels of antibodies in HIV-infected and uninfected individuals at a single timepoint without longitudinal follow up and this makes it difficult in interpreting their implication with respect to malaria protection or vulnerability. Further, this study examined antibody concentration prior to antiretroviral treatment and using malaria antigens (AMA-1 and GLURP-R0) are highly polymorphic. The impact of HIV treatment on subclass destruction should be examined and additional malaria antigen should be evaluated. Future studies should also investigate the cellular mechanisms leading to elevated antibody levels in HIV-infected individuals and whether this elevation influences the affinity and effector function of antibodies produced targeting non-HIV antigens such as malaria.

## Supplementary information

**Additional file 1: Fig. S1.** Sample scatter plots of AMA1 antibody concentrations versus against VL and CRP. **Fig. S2.** IgG3:IgG1 ratio for AMA1 and GLURP specific antibodies by CD4 counts. **Table S1.** Inter-assay and Intra-assay CV for IgM and IgG subclasses.

## Data Availability

The datasets used for the current study are available on reasonable request. However, research conducted through the Kenyan Medical Research Institute requires approval. Requests to access the datasets should be directed to the corresponding author, Dr. Anne Frosch who request approval through the Kenyan Medical Research Institute.

## Abbreviations

AMA1: apical membrane antigen-1
AU: arbitrary units
BCR: B cell receptor
CD4: cluster of differentiation 4
CRP: C-reactove protein
GLURP-R0: glutamate rich protein-R0
HIV: human immunodeficiency virus
IFN: interferon
IQR: interquartile range
OD: optical density
TCR: T cell receptor
TLR9: Toll-like receptor 9
Tfh: T follicular helper cells
VL: viral load

## References

[1] Moir S, Fauci AS (2009). B cells in HIV infection and disease. Nat Rev Immunol.

[2] Carson PJ, Schut RL, Simpson ML, O’Brien J, Janoff EN (1995). Antibody class and subclass responses to pneumococcal polysaccharides following immunization of human immunodeficiency virus-infected patients. J Infect Dis.

[3] Johannesson TG, Sogaard OS, Tolstrup M, Petersen MS, Bernth-Jensen JM, Ostergaard L (2012). The impact of B-cell perturbations on pneumococcal conjugate vaccine response in HIV-infected adults. PLoS ONE.

[4] Madhi SA, Petersen K, Madhi A, Khoosal M, Klugman KP (2000). Increased disease burden and antibiotic resistance of bacteria causing severe community-acquired lower respiratory tract infections in human immunodeficiency virus type 1-infected children. Clin Infect Dis.

[5] Malaspina A, Moir S, Orsega SM, Vasquez J, Miller NJ, Donoghue ET (2005). Compromised B cell responses to influenza vaccination in HIV-infected individuals. J Infect Dis.

[6] Titanji K, De Milito A, Cagigi A, Thorstensson R, Grutzmeier S, Atlas A (2006). Loss of memory B cells impairs maintenance of long-term serologic memory during HIV-1 infection. Blood.

[7] Cubas RA, Mudd JC, Savoye AL, Perreau M, van Grevenynghe J, Metcalf T (2013). Inadequate T follicular cell help impairs B cell immunity during HIV infection. Nat Med.

[8] Malaspina A, Moir S, Kottilil S, Hallahan CW, Ehler LA, Liu S (2003). Deleterious effect of HIV-1 plasma viremia on B cell costimulatory function. J Immunol..

[9] Moir S, Ho J, Malaspina A, Wang W, DiPoto AC, O’Shea MA (2008). Evidence for HIV-associated B cell exhaustion in a dysfunctional memory B cell compartment in HIV-infected viremic individuals. J Exp Med.

[10] Moir S, Ogwaro KM, Malaspina A, Vasquez J, Donoghue ET, Hallahan CW (2003). Perturbations in B cell responsiveness to CD4^+^ T cell help in HIV-infected individuals. Proc Natl Acad Sci USA.

[11] Pallikkuth S, Parmigiani A, Silva SY, George VK, Fischl M, Pahwa R (2012). Impaired peripheral blood T-follicular helper cell function in HIV-infected non-responders to the 2009 H1N1/09 vaccine. Blood.

[12] Frosch AE, Odumade OA, Taylor JJ, Ireland K, Ayodo G, Ondigo B (2017). Decrease in numbers of naive and resting B cells in HIV-infected Kenyan adults leads to a proportional increase in total and *Plasmodium falciparum*-specific atypical memory B Cells. J Immunol..

[13] Murray CJ, Ortblad KF, Guinovart C, Lim SS, Wolock TM, Roberts DA (2014). Global, regional, and national incidence and mortality for HIV, tuberculosis, and malaria during 1990–2013: a systematic analysis for the Global Burden of Disease Study 2013. Lancet.

[14] Polyak CS, Yuhas K, Singa B, Khaemba M, Walson J, Richardson BA (2016). Cotrimoxazole prophylaxis discontinuation among antiretroviral-treated HIV-1-infected adults in Kenya: a randomized non-inferiority trial. PLoS Med..

[15] Abu-Raddad LJ, Patnaik P, Kublin JG (2006). Dual infection with HIV and malaria fuels the spread of both diseases in sub-Saharan Africa. Science.

[16] Herrero MD, Rivas P, Rallon NI, Ramirez-Olivencia G, Puente S (2007). HIV and malaria. AIDS Rev..

[17] Gonzalez R, Ataide R, Naniche D, Menendez C, Mayor A (2012). HIV and malaria interactions: where do we stand?. Expert Rev Anti Infect Ther..

[18] Whitworth J, Morgan D, Quigley M, Smith A, Mayanja B, Eotu H (2000). Effect of HIV-1 and increasing immunosuppression on malaria parasitaemia and clinical episodes in adults in rural Uganda: a cohort study. Lancet.

[19] John CC, Tande AJ, Moormann AM, Sumba PO, Lanar DE, Min XM (2008). Antibodies to pre-erythrocytic *Plasmodium falciparum* antigens and risk of clinical malaria in Kenyan children. J Infect Dis.

[20] Nebie I, Diarra A, Ouedraogo A, Soulama I, Bougouma EC, Tiono AB (2008). Humoral responses to *Plasmodium falciparum* blood-stage antigens and association with incidence of clinical malaria in children living in an area of seasonal malaria transmission in Burkina Faso, West Africa. Infect Immun..

[21] Sabchareon A, Burnouf T, Ouattara D, Attanath P, Bouharoun-Tayoun H, Chantavanich P (1991). Parasitologic and clinical human response to immunoglobulin administration in falciparum malaria. Am J Trop Med Hyg.

[22] Hasang W, Dembo EG, Wijesinghe R, Molyneux ME, Kublin JG, Rogerson S (2014). HIV-1 infection and antibodies to P*lasmodium falciparum* in adults. J Infect Dis.

[23] Mount AM, Mwapasa V, Elliott SR, Beeson JG, Tadesse E, Lema VM (2004). Impairment of humoral immunity to *Plasmodium falciparum* malaria in pregnancy by HIV infection. Lancet.

[24] Nnedu ON, O’Leary MP, Mutua D, Mutai B, Kalantari-Dehaghi M, Jasinskas A (2011). Humoral immune responses to *Plasmodium falciparum* among HIV-1-infected Kenyan adults. Proteomics Clin Appl..

[25] Nimmerjahn F, Ravetch JV (2008). Fcgamma receptors as regulators of immune responses. Nat Rev Immunol.

[26] Noland GS, Jansen P, Vulule JM, Park GS, Ondigo BN, Kazura JW (2015). Effect of transmission intensity and age on subclass antibody responses to *Plasmodium falciparum* pre-erythrocytic and blood-stage antigens. Acta Trop.

[27] Pattanapanyasat K, Sukapirom K, Kowawisatsut L, Thepthai C (2008). New BD FACSCount CD4 reagent system for simultaneous enumeration of percent and absolute CD4 T-lymphocytes in HIV-1-infected pediatric patients. Cytometry B Clin Cytom..

[28] Marconi A, Balestrieri M, Comastri G, Pulvirenti FR, Gennari W, Tagliazucchi S (2009). Evaluation of the Abbott Real-Time HIV-1 quantitative assay with dried blood spot specimens. Clin Microbiol Infect.

[29] Tang N, Huang S, Salituro J, Mak WB, Cloherty G, Johanson J (2007). A RealTime HIV-1 viral load assay for automated quantitation of HIV-1 RNA in genetically diverse group M subtypes A-H, group O and group N samples. J Virol Methods.

[30] Lasselin J, Laye S, Dexpert S, Aubert A, Gonzalez C, Gin H (2012). Fatigue symptoms relate to systemic inflammation in patients with type 2 diabetes. Brain Behav Immun.

[31] Ryberg H, An J, Darko S, Lustgarten JL, Jaffa M, Gopalakrishnan V (2010). Discovery and verification of amyotrophic lateral sclerosis biomarkers by proteomics. Muscle Nerve.

[32] Barry AE, Arnott A (2014). Strategies for designing and monitoring malaria vaccines targeting diverse antigens. Front Immunol..

[33] Esen M, Kremsner PG, Schleucher R, Gassler M, Imoukhuede EB, Imbault N (2009). Safety and immunogenicity of GMZ2—a MSP3-GLURP fusion protein malaria vaccine candidate. Vaccine..

[34] Iriemenam NC, Khirelsied AH, Nasr A, ElGhazali G, Giha HA, Elhassan AETM (2009). Antibody responses to a panel of *Plasmodium falciparum* malaria blood-stage antigens in relation to clinical disease outcome in Sudan. Vaccine..

[35] Jepsen MP, Jogdand PS, Singh SK, Esen M, Christiansen M, Issifou S (2013). The malaria vaccine candidate GMZ2 elicits functional antibodies in individuals from malaria endemic and non-endemic areas. J Infect Dis.

[36] Mordmuller B, Szywon K, Greutelaers B, Esen M, Mewono L, Treut C (2010). Safety and immunogenicity of the malaria vaccine candidate GMZ2 in malaria-exposed, adult individuals from Lambarene, Gabon. Vaccine..

[37] Thera MA, Doumbo OK, Coulibaly D, Laurens MB, Kone AK, Guindo AB (2010). Safety and immunogenicity of an AMA1 malaria vaccine in Malian children: results of a phase 1 randomized controlled trial. PLoS ONE.

[38] Chelimo K, Ofulla AV, Narum DL, Kazura JW, Lanar DE, John CC (2005). Antibodies to *Plasmodium falciparum* antigens vary by age and antigen in children in a malaria-holoendemic area of Kenya. Pediatr Infect Dis J..

[39] John CC, Moormann AM, Pregibon DC, Sumba PO, McHugh MM, Narum DL (2005). Correlation of high levels of antibodies to multiple pre-erythrocytic *Plasmodium falciparum* antigens and protection from infection. Am J Trop Med Hyg.

[40] Kariuki SK, Lal AA, Terlouw DJ, ter Kuile FO, Ong’echa JM, Phillips-Howard PA (2003). Effects of permethrin-treated bed nets on immunity to malaria in western Kenya II. Antibody responses in young children in an area of intense malaria transmission. Am J Trop Med Hyg..

[41] Noland GS, Hendel-Paterson B, Min XM, Moormann AM, Vulule JM, Narum DL (2008). Low prevalence of antibodies to preerythrocytic but not blood-stage *Plasmodium falciparum* antigens in an area of unstable malaria transmission compared to prevalence in an area of stable malaria transmission. Infect Immun.

[42] Akoglu H (2018). User’s guide to correlation coefficients. Turk J Emerg Med..

[43] Hochman S, Kim K (2009). The impact of HIV and malaria coinfection: what is known and suggested venues for further study. Interdiscip Perspect Infect Dis..

[44] Kamya MR, Gasasira AF, Yeka A, Bakyaita N, Nsobya SL, Francis D (2006). Effect of HIV-1 infection on antimalarial treatment outcomes in Uganda: a population-based study. J Infect Dis.

[45] Groux H, Gysin J (1990). Opsonization as an effector mechanism in human protection against asexual blood stages of *Plasmodium falciparum*: functional role of IgG subclasses. Res Immunol.

[46] Roussilhon C, Oeuvray C, Muller-Graf C, Tall A, Rogier C, Trape JF (2007). Long-term clinical protection from falciparum malaria is strongly associated with IgG3 antibodies to merozoite surface protein 3. PLoS Med..

[47] Taylor RR, Allen SJ, Greenwood BM, Riley EM (1998). IgG3 antibodies to *Plasmodium falciparum* merozoite surface protein 2 (MSP2): increasing prevalence with age and association with clinical immunity to malaria. Am J Trop Med Hyg.

[48] Pleass RJ, Moore SC, Stevenson L, Hviid L (2016). Immunoglobulin M: restrainer of inflammation and mediator of immune evasion by *Plasmodium falciparum* malaria. Trends Parasitol..

[49] Subramaniam KS, Skinner J, Ivan E, Mutimura E, Kim RS, Feintuch CM (2015). HIV malaria co-infection is associated with atypical memory B cell expansion and a reduced antibody response to a broad array of *Plasmodium falciparum* antigens in Rwandan Adults. PLoS ONE.

[50] Okech B, Mujuzi G, Ogwal A, Shirai H, Horii T, Egwang TG (2006). High titers of IgG antibodies against *Plasmodium falciparum* serine repeat antigen 5 (SERA5) are associated with protection against severe malaria in Ugandan children. Am J Trop Med Hyg.

[51] Polley SD, Conway DJ, Cavanagh DR, McBride JS, Lowe BS, Williams TN (2006). High levels of serum antibodies to merozoite surface protein 2 of *Plasmodium falciparum* are associated with reduced risk of clinical malaria in coastal Kenya. Vaccine..

[52] Langhorne J, Ndungu FM, Sponaas AM, Marsh K (2008). Immunity to malaria: more questions than answers. Nat Immunol.

[53] Bartmann P, Grosch-Worner I, Wahn V, Belohradsky BH (1991). IgG2 deficiency in children with human immunodeficiency virus infection. Eur J Pediatr.

[54] Nunes MC, von Gottberg A, de Gouveia L, Cohen C, Kuwanda L, Karstaedt AS (2011). Persistent high burden of invasive pneumococcal disease in South African HIV-infected adults in the era of an antiretroviral treatment program. PLoS ONE.

[55] Wall EC, Cartwright K, Scarborough M, Ajdukiewicz KM, Goodson P, Mwambene J (2013). High mortality amongst adolescents and adults with bacterial meningitis in sub-Saharan Africa: an analysis of 715 cases from Malawi. PLoS ONE.

[56] Obeng-Adjei N, Portugal S, Holla P, Li S, Sohn H, Ambegaonkar A (2017). Malaria-induced interferon-gamma drives the expansion of Tbethi atypical memory B cells. PLoS Pathog.

[57] Lindqvist M, van Lunzen J, Soghoian DZ, Kuhl BD, Ranasinghe S, Kranias G (2012). Expansion of HIV-specific T follicular helper cells in chronic HIV infection. J Clin Invest..

[58] Kohler SL, Pham MN, Folkvord JM, Arends T, Miller SM, Miles B (2016). Germinal center T follicular helper cells are highly permissive to HIV-1 and alter their phenotype during virus replication. J Immunol..

[59] Velu V, Mylvaganam G, Ibegbu C, Amara RR (2018). Tfh1 cells in germinal centers during chronic HIV/SIV infection. Front Immunol..

[60] Gomez AM, Ouellet M, Tremblay MJ (2015). HIV-1-triggered release of type I IFN by plasmacytoid dendritic cells induces BAFF production in monocytes. J Immunol..

[61] Chung NP, Matthews K, Klasse PJ, Sanders RW, Moore JP (2012). HIV-1 gp120 impairs the induction of B cell responses by TLR9-activated plasmacytoid dendritic cells. J Immunol..

