# Supplementary material for: HIV infection drives IgM and IgG3 subclass bias in Plasmodium falciparum-specific and total immunoglobulin concentration in Western Kenya
Source: Malar J. 2019 Aug 30;18:297. doi: 10.1186/s12936-019-2915-7 (PMC6716850; doi:10.1186/s12936-019-2915-7)
Supplement: Supplementary file 1 — Additional file 1: Fig. S1. Sample scatter plots of AMA1 antibody concentrations versus against VL and CRP. Fig. S2. IgG3:IgG1 ratio for AMA1 and GLURP specific antibodies by CD4 counts. Table S1. Inter-assay and Intra-assay CV for IgM and IgG subclasses. [file 12936_2019_2915_MOESM1_ESM.pdf]

Additional Files:

Figure S1: Sample scatter plots of AMA1 antibody concentrations versus against VL and CRP.

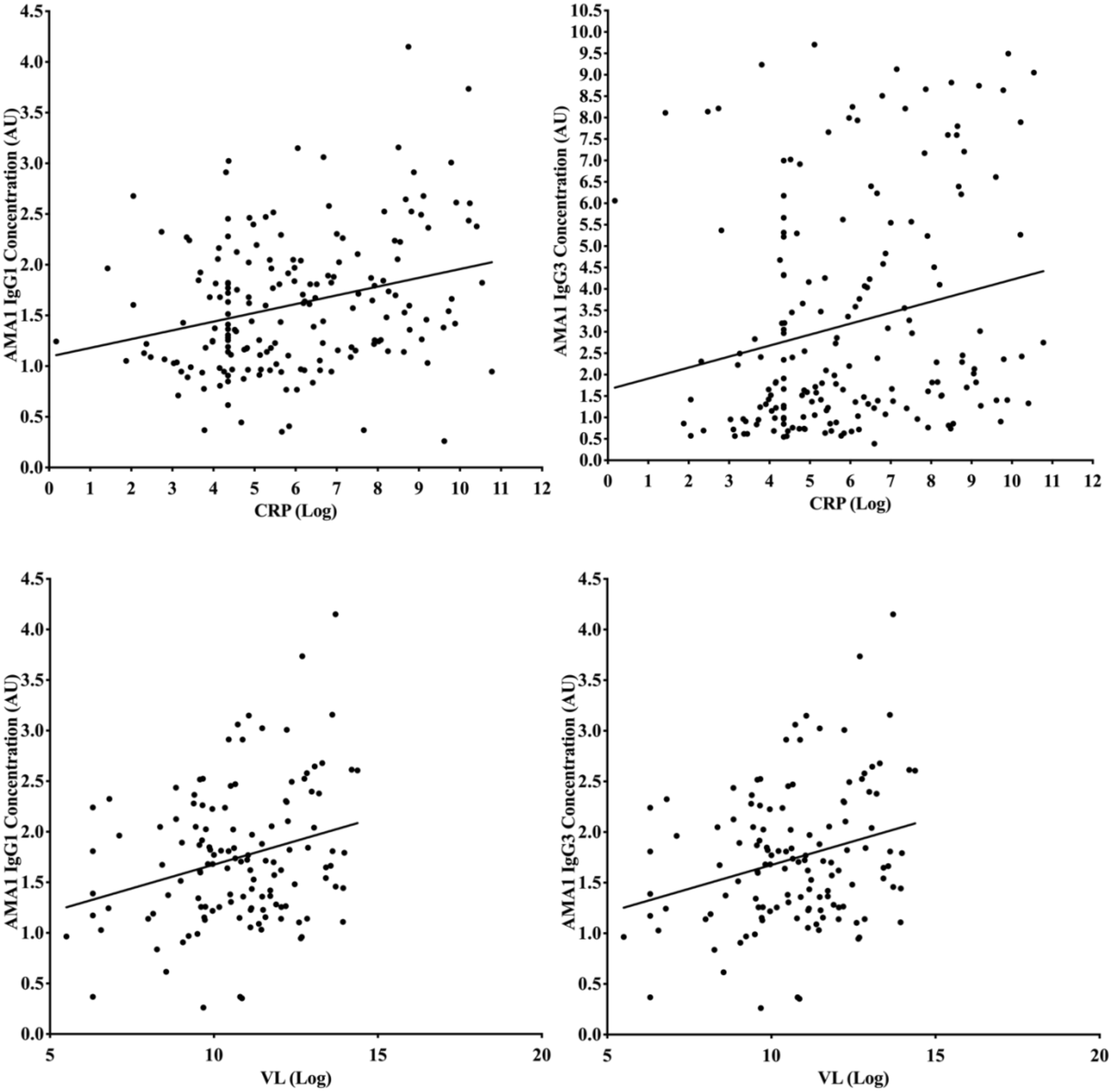

Figure S2: IgG3: IgG1 ratio for AMA1 and GLURP specific antibodies by CD4 counts.

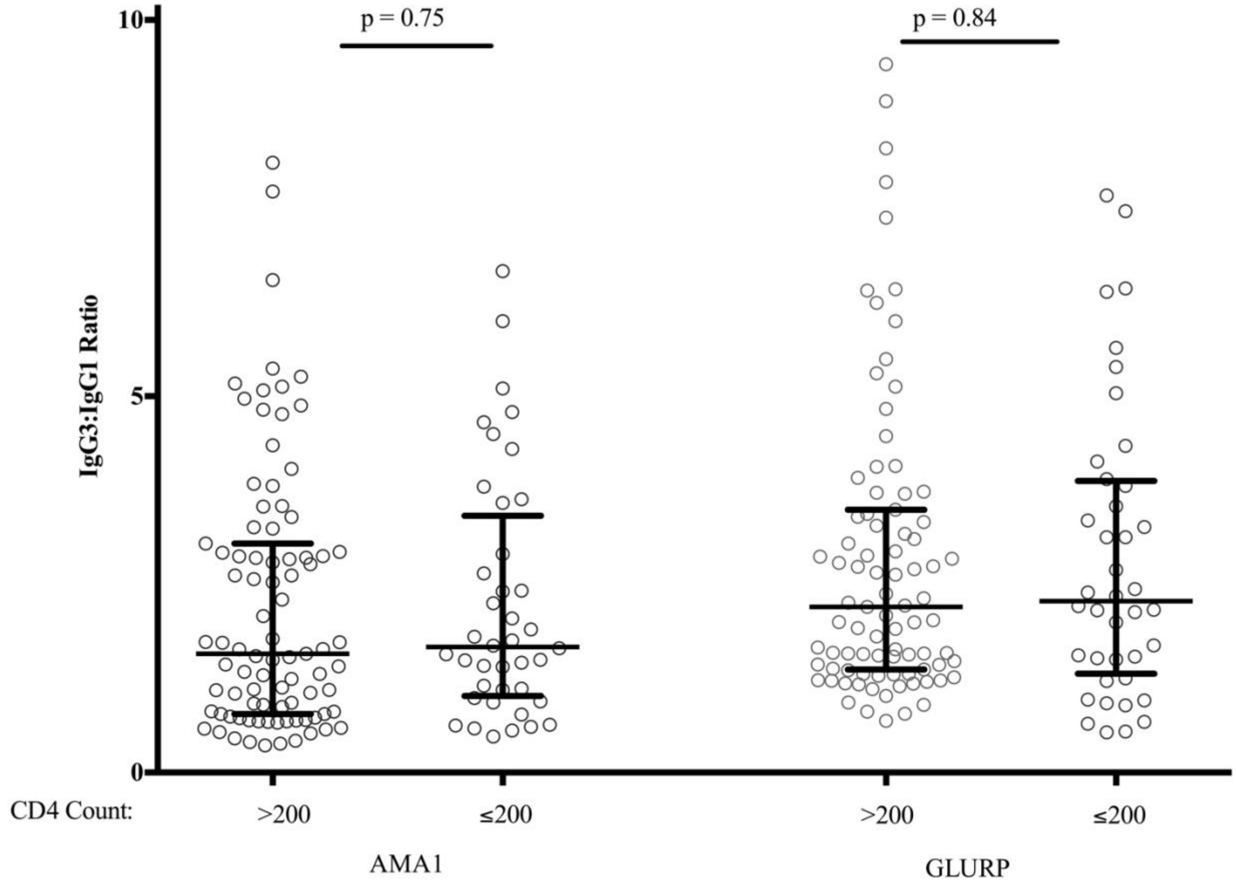

Table S1: Inter-assay and Intra-assay CV for IgM and IgG subclasses.

Antigen	Class/ Subclass	N	Inter-assay CV (%)	N	Intra-assay CV (%)
AMA 1	IgG1	23	5.31	12	3.40
	IgG2	23	8.19	12	8.04
	IgG3	23	5.99	12	4.72
	IgG4	23	10.03	12	4.80
	IgM	23	10.00	12	4.03
GLURP-RO	IgG1	23	18.73	12	18.83
	IgG2	23	10.00	12	6.10
	IgG3	23	15.83	12	5.46
	IgG4	23	8.09	12	8.64
	IgM	23	7.26	12	1.88
Minimum			5.31		1.88
Maximum			18.73		18.83
Median			9.09		5.13
